# Rutin Inhibits *Streptococcus suis* Biofilm Formation by Affecting CPS Biosynthesis

**DOI:** 10.3389/fphar.2017.00379

**Published:** 2017-06-16

**Authors:** Shuai Wang, Chang Wang, Lingfei Gao, Hua Cai, Yonghui Zhou, Yanbei Yang, Changgeng Xu, Wenya Ding, Jianqing Chen, Ishfaq Muhammad, Xueying Chen, Xinmiao He, Di Liu, Yanhua Li

**Affiliations:** ^1^College of Veterinary Medicine, Northeast Agricultural UniversityHarbin, China; ^2^Heilongjiang Key Laboratory for Animal Disease Control and Pharmaceutical DevelopmentHarbin, China; ^3^Harbin Pharmaceutical Group Bio-Vaccine Co. Ltd. (Hayao Vaccine),Harbin, China; ^4^Heilongjiang Key Laboratory for Animal Disease Control and Pharmaceutical Development, Northeast Agricultural UniversityHarbin, China

**Keywords:** *Streptococcus suis*, biofilms, rutin, adhesion, CPS

## Abstract

*Streptococcus suis* (*S. suis*) form biofilms and causes severe diseases in humans and pigs. Biofilms are communities of microbes embedded in a matrix of extracellular polymeric substances. Eradicating biofilms with the use of antibiotics or biocides is often ineffective and needs replacement with other potential agents. Compared to conventional agents, promising and potential alternatives are biofilm-inhibiting compounds without impairing growth. Here, we screened a *S. suis* adhesion inhibitor, rutin, derived from *Syringa*. Rutin, a kind of flavonoids, shows efficient biofilm inhibition of *S. suis* without impairing its growth. Capsular polysaccharides(CPS) are reported to be involved in its adherence to influence bacterial biofilm formation. We investigated the effect of rutin on *S. suis* CPS content and structure. The results showed that rutin was beneficial to improve the CPS content of *S. suis* without changing its structure. We further provided evidence that rutin specifically affected *S. suis* biofilm susceptibility by affecting CPS biosynthesis *in vitro*. The study explores the antibiofilm potential of rutin against *S. suis* which can be used as an adhesion inhibitor for the prevention of *S. suis* biofilm-related infections. Nevertheless, rutin could be used as a novel natural inhibitor of biolfilm and its molecular mechanism provide basis for its pharmacological and clinical applications.

## Introduction

*Streptococcus suis* (*S. suis*) is a major swine pathogen and a zoonotic agent that causes severe invasive diseases in pigs, including meningitis and streptococcal toxic shock-like syndrome (Gottschalk et al., 2010). What’s more, *S. suis* is a major public health issue and an emerging zoonotic agent in Southeast and East Asia (Sriskandan and Slater, 2006; Gottschalk et al., 2007). In 2005, a survey showed more than two hundred human cases of *S. suis* in China among them 39 were found dead (Yang et al., 2006). Meanwhile, studies showed that *S. suis* could cause persistent infections due to its ability of forming biofilms *in vivo* (Wang Y. et al., 2011).

Biofilms are microbial sessile communities characterized by bacterium that are adhered to biotic or abiotic surfaces or to each other, are surrounded by a polymer matrix and exhibit an altered phenotype compared to planktonic cells (Fux et al., 2005). Biofilm cells are known to be 10–1,000 times more opposed to antimicrobial agents compared to planktonic cells (Gilbert et al., 1997). This may be due to a decreased penetration of antibiotics, a decreased growth rate of the biofilm cells and/or a decreased metabolism of bacterial cells in biofilms (Kiedrowski and Horswill, 2011). In addition, the presence of persisted cells and the expression of specific resistance genes in biofilms may contribute to this tolerance (Conlon, 2014).

Bacterial adhesion to a substrate is the first essential step for biofilm formation. Meanwhile, it has been reported that CPS was involved in the adherence of *pneumococci* to host cells (Allegrucci and Sauer, 2007), and the encapsulated isolated clinical *pneumococcal* were found to have impaired biofilm formation (Moscoso et al., 2006). Previously, several studies have demonstrated that the mutant strain of non-encapsulated *pneumococcal* showed stronger adhesion ability and enhanced biofilm formation ability than their encapsulated parents *in vitro* (Waite et al., 2001; Moscoso et al., 2006; Allegrucci and Sauer, 2007; McEllistrem et al., 2007). It is well known that Wzx/Wzy-dependent pathway is responsible for the formation of *S. suis* CPS (Wang K. et al., 2011). In this pathway, first, an initial monosaccharide is linked to the inner face of the cytoplasmic membrane by an initial sugar transferase. Second, other monosaccharides are joined sequentially by specific glycosyltransferases to assemble repeated units. Then, the repeated units are transported to the outer surface of the cytoplasmic membrane by Wzx flippase, and each repeated unit is combined and polymerized to form the lipid linked CP by Wzy polymerase. Finally, mature CPS is translocated to the peptidoglycan by the membrane protein complex (Okura et al., 2013). The genes involved in this pathway contain a gene cluster (*cps* gene cluster) and are usually located at the same chromosomal locus (Bentley et al., 2006). The *cps* gene cluster includes the genes encoding the initial sugar transferase, Wzx flippase (*wzx*), Wzy polymerase (*wzy*), additional glycosyltransferases, and enzymes to modify the repeated units or to add other moieties on CPS (Roberts, 1996; Bentley et al., 2006; Okura et al., 2013). Therefore, adhesion inhibitors were screened by affecting CPS biosynthesis.

Recently, few novel bactericidal or bacteriostatic agents have been developed and their antimicrobial activity lead to selective pressure, with antimicrobial resistance as an inevitable consequence of their use (Roca et al., 2015). For this reason, innovative antimicrobials with novel targets and modes of action are needed. A recently developed method aimed at interference with biofilm development without affecting bacterial growth compared to traditional bactericidal or bacteriostatic uses to inhibit biofilms (McDougald et al., 2012). Various natural products are successful in regulating biofilms development. The benefits of using natural products in biofilm inhibition are their higher specificity and lower toxicity compared to synthetic compounds (Koo and Jeon, 2009). For example, mulberry leaves has been shown to inhibit *Streptococcus mutans* biofilm formation by affecting bacterial adhesion (Islam et al., 2008).

Rutin, a well-known and widely used citrus flavonoid glycoside, has a lot of benefical pharmacological effects such as antimicrobial, antioxidant, anti-inflammatory, and antihypertensive effects (Erlund et al., 2000; Middleton et al., 2000). It is found in many foods, such as orange, buckwheat, apple, onion, lemon, and grapefruit. In addition, rutin was identified as the principal anti-biofilm compounds in burdock leaf and inhibited biofilm formation of *Pseudomonas aeruginosa* by betabolomics-based screening (Lou et al., 2015). We have analyzed the relationship between the spectrum and the impact of *Syringa oblata Lindl.* aqueous extract on *S. suis* biofilms *in vitro.* According to high performance liquid chromatography (HPLC) fingerprint and anti-biofilm activity test, gray relational analysis was applied to find the active composition. Rutin make significant contribution to anti-biofilm activity according to the relational grade analysis. Our previous results also showed that rutin was confirmed as the main anti-biofilm compounds in *Syringa oblata Lindl.* aqueous extract and truly affects *S. suis* biofilms *in vitro* (Bai et al., 2017).

In this work, we provided evidence that rutin inhibits the biofilm formation of *S. suis* by affecting CPS biosynthesis *in vitro*. The study explores the antibiofilm potential of rutin against *S. suis* which can be used as an adhesion inhibitor for the prevention of *S. suis* biofilm-related infections.

## Materials and Methods

### Bacterial Strains and Growth Conditions

*Streptococcus suis* (ATCC700794) was purchased from the American Type Culture Collection. Bacteria were cultured aerobically as mentioned in our previous study (Yang et al., 2015). Briefly, bacteria grown at 37°C in Todd-Hewitt broth (THB; Sigma–Aldrich) or Todd-Hewitt broth agar (THA) added with 5% (v/v) fetal bovine serum (Sijiqing Ltd, Hangzhou, China) (Yang et al., 2015).

### Effectiveness of Rutin on Inhibition of Biofilm by the TCP Assay

The effect of rutin on *S. suis* biofilm formation was assayed as previously described (Bai et al., 2017). Briefly, a mid-exponential growth culture of *S. suis* was diluted to an optical density of 10^5^–10^6^ cells/mL. Then, 100 μL of cultures distributed in 96-well microplates (Corning Costar, NY, United States) containing 100 μL of sub-MICs of rutin solution (1/2 MIC (0.1563 mg/mL), 1/4 MIC (0.0781 mg/mL), 1/8 MIC (0.0391 mg/mL), and 1/16 MIC (0.0195 mg/mL)), negative control wells were included. After incubation for 72 h at 37°C without shaking, the wells were washed twice with distilled water, dried and fixed with 200 μL of 99% methanol for 5 min. Then, the wells were decanted, left to dry and stained with 200 μL of 2% crystal violet for another 5 min. Excess stain was rinsed off gently by water, the microplates were air dried, and the bound dye was solubilized with 200 μL of 33% glacial acetic acid. Crystal violet-stained bacteria were quantified measuring the A595 nm (DG5033A, Huadong Ltd, Nanjing, China). The reported values are the means of three measurements (Bai et al., 2017).

### Growth Inhibitory Test

Growth inhibitory test was performed by the previously described method with minor modifications (Yang et al., 2015). Briefly, the culture medium was added with 1/4 MIC (0.0781 mg/mL) of rutin and incubated at 37°C for 24 h. Control cells were also incubated in the absence of rutin. The rutin-treated and untreated cultures samples were taken every hour for measuring at OD_600__nm_.

### Observation by Scanning Electron Microscopy (SEM)

A mid-exponential growth culture of *S. suis* was diluted to an optical density of 10^5^–10^6^ cells/mL. Then, a volume of 2 mL was added to a 6-well microplates (Corning Costar, NY, United States) containing a sterilized rough glass slide (1 cm × 1 cm). After culturing in stationary conditions for 72 h at 37°C, the glass slide were rinsed with sterile PBS in order to eliminate any non-adherent bacteria. The remaining biofilms were fixed with fixative solution (2 mM CaCl_2_ in 0.2 M cacodylate buffer, 2.5% (w/v) glutaraldehyde, 4% (w/v) paraformaldehyde, pH 7.2) for 6 h and washed with PBS, then fixed in 2% osmium tetroxide containing 6% (w/v) sucrose and 2 mM potassium ferrocyanide in cacodylate buffer. The samples were dried, gold sputtered with an ion sputtering instrument (current 15 mA, 2 min) and observed using SEM (FEI Quanta, Netherland) (Bai et al., 2017).

### Adhesion Assays

Adhesion assays was performed as previously described (Hamada et al., 1981). In brief, a mid-exponential growth culture of *S. suis* was diluted to an optical density of 10^5^–10^6^ cells/mL and each 2 mL [THB or 1/4MIC (0.0781 mg/mL) rutin] were added to a 6-well microplate (Corning Costar, NY, United States) containing sterilized rough organic membrane (1 cm × 1 cm). The plate were incubated in stationary conditions for for 24 h at 37°C, the non-adherent bacteria were decanted, and the remaining adherence were removed by 0.5 M of sodium hydroxide. Then, the cells were quantified at 600 nm. Percentage adherence = [OD_600_ of adhered cells/ (OD_600_ of adhered cells+OD_600_ of supernatant cells)].

### PCR Amplification and Nucleotide Sequencing

To investigate whether rutin cause the *cps* gene mutation, a mid-exponential growth culture of *S. suis* was diluted to an optical density of 10^5^–10^6^ cells/mL and the culture medium was supplemented with 1/4 MIC (0.0781mg/mL) of rutin incubating at 37°C for 24 h. Control cells were also incubated in the absence of rutin. Chromosomal DNA from *S. suis* was prepared as previously described (Qin et al., 2006). Briefly, Bacterial colonies were suspended in 50 μL distilled water to extract DNA, followed by boiling for 5 min. Ex Taq DNA polymerase was used to perform PCR choosing the conditions: 94°C for 10 min, 35 cycles of 94°C for 30 s, 57°C–60°C for 30 s (Details are shown in **Table 1**), and 72°C for 30 s; followed by a final extension step at 72°C for 10 min. The following primer pairs used in this work are listed in **Table 1**. The identity of the PCR product was confirmed by DNA sequencing. Sequence alignments were conducted using the BLAST program.

**Table 1:** Primers used for the PCR.

Genes	Primer sequence	Annealing temperature
*cps 2A*	Forward: 5′- TTCCACCCACCAAGTCGA -3′	60°C
	Reverse: 5′- TGGCGGGCAAAATCAATA -3′	
*cps 2B*	Forward: 5′- TCTATTTCTGTGCGTGAT -3′	60°C
	Reverse: 5′- TTGGAGTGGTTGGTTCTT -3′	
*cps 2C*	Forward: 5′- ATAACCTGAACGAGCATA -3′	57°C
	Reverse: 5′- AGAGAGGGAGTAAATAAAAC -3′	
*cps 2D*	Forward: 5′- GATTTTTTCTGGTGTTTC -3′	60°C
	Reverse: 5′- TCATATTTGGTGTGGATG -3′	
*cps 2E*	Forward: 5′- TGTCCATTTTGAACATCC -3′	60°C
	Reverse: 5′- TTTTGCTCAGAAACGAGT -3′	
*cps 2F*	Forward: 5′- AGGACCAATCCGACAAGC -3′	60°C
	Reverse: 5′- TGAACATAATGGAGCAAC -3′	
*cps 2G*	Forward: 5′- CATACCAATGACAAGAGC -3′	60°C
	Reverse: 5′- GACCAAATCAGTGTAATCTA -3′	
*cps 2H*	Forward: 5′- GCCTCTTATTCAGGTTAT -3′	60°C
	Reverse: 5′- CTTTTCTTTTTGTTTTCG -3′	
*cps2I*	Forward: 5′- GTACTCCATTGTCTTTGT -3′	57°C
	Reverse: 5′- GAATTGTTTTTGATTCTT -3′	
*cps 2J*	Forward: 5′- ACCGCTCATATAATGATT -3′	57°C
	Reverse: 5′- GGAGGGTTACTTGCTACT -3′	
*cps 2K*	Forward: 5′- CCAGCAACTGCCACAAGG -3′	57°C
	Reverse: 5′- GCATAAGTCGCGCCAAGG -3′	
*cps 2L*	Forward: 5′- CAGAAAGCAACAGAAAAA -3′	60°C
	Reverse: 5′- GAATCCAACAAATAGTAGAATA -3′	
*cps 2M*	Forward: 5′- AACAGGCAAATTAGAAAG -3′	60°C
	Reverse: 5′- TGATAAAGTATGGACAGAAG -3′	
*cps 2N*	Forward: 5′- CTCGTGGGTGCGGTTTTA -3′	60°C
	Reverse: 5′- CAGCCCTTGTCTTGGGATTA -3′	
*cps 2O*	Forward: 5′- CGATAGGGGCTGACTGAG -3′	57°C
	Reverse: 5′- CGCGAGAAATTTGATGA -3′	
*cps 2P*	Forward: 5′- AAGCAGGGTAAGGGGTTG -3′	60°C
	Reverse: 5′- ACTGGTATGGCTGTTATGGA -3′	
*cps 2Q*	Forward: 5′- CCGCTTCGATGTCTTTGA -3′	60°C
	Reverse: 5′- TGGGATTATGCGTCGCTTAT -3′	
*cps 2R*	Forward: 5′- TATAGTGACGAAGACAGC -3′	60°C
	Reverse: 5′-ATATTTTGAAGATAAACCG -3′	
*cps 2S*	Forward: 5′-AACAAGGCTAACAGACGA -3′	60°C
	Reverse: 5′-GTGATGACGAAGGAAGAT -3′	
*cps 2T*	Forward: 5′-GCAACTAAAAATAAATTG -3′	57°C
	Reverse: 5′-AAGAGTGCTCTAAGACCA -3′	
*cps 2U*	Forward: 5′-ATACCTCGCCATCTTTTA -3′	60°C
	Reverse: 5′-GCTGATTCCCTCTTTTTG -3′	

### RNA Isolation and Quantitative RT-PCR

The quantitative Real-Time PCR was performed as described in our previous study (Yang et al., 2015). To investigate the effect of rutin on expression of *cps* gene, a mid-exponential growth culture of *S. suis* was diluted to an optical density of 10^5^–10^6^ cells/mL and the culture medium was added with 1/4 MIC (0.0781mg/mL) of rutin prior to further incubating at 37°C for 24 h. The control cells were also incubated without rutin. Bacteria were collected by centrifugation (12,000 ×*g*, 5 min, 4°C) and treated with an RNASE REMOVER I (Huayueyang Ltd, Beijing, China). RNA extraction was performed with an E.Z.N.A. Bacterial RNA isolating kit (Omega, Beijing, China) following the manufacturer’s instructions. RNA (100 ng/mL) was reverse transcribed in S1000 thermal cycler (Bio-Rad Laboratories) by using Moloney murine leukemia virus reverse transcriptase and random hexamers. The specific primers used for the quantitative RT-PCR (listed in **Table 2**) were designed by Takara Company (Takara Ltd, Dalian, Liaoning, China). The 16S rRNA gene was used for normalization of target genes. The amplification conditions for *cps* and 16S rRNA were 94°C for 10 min followed by 30 cycles at 94°C for 15 s, 60°C for 60 s and 72°C for 20 s, and then 72°C for 10 min (Yang et al., 2015).

**Table 2:** Primers used for the quantitative RT-PCR analysis.

Genes	Primer sequence
*cps 2A*	Forward: 5′-ATGAAAAAGAGAAGCGGACGAAGTA-3′
	Reverse: 5′- TTATTTTTCAACAAGTACGGACTGA-3′
*cps 2B*	Forward: 5′-ATGAACAATCAAGAAGTAAATGCAA-3′
	Reverse: 5′- CTATTTTAATTTCTTCGAATCTGGT-3′
*cps 2C*	Forward: 5′-ATGGCGATGTTAGAAATTGCACGTA-3′
	Reverse: 5′- TTAGGCTTTTTTGCCGTAATTTCCG-3′
*cps 2D*	Forward: 5′-ATGATTGATATCCATTCGCATATCA-3′
	Reverse: 5′- TTACTGTACTTGATTTTTCAATATC-3′
*cps 2E*	Forward: 5′-ATGAATATTGAAATAGGATATCGCC-3′
	Reverse: 5′- TTACTTACTTCCCTCTCTCAACAAT-3′
*cps 2F*	Forward: 5′-ATGAGAACAGTTTATATTATTGGTT-3′
	Reverse: 5′- TTATCCTTTAAACAACTTCTCATAC-3′
*cps 2G*	Forward: 5′-ATGAAAAAGATTCTATATCTCCATG-3′
	Reverse: 5′- TCAGTATACTTTGAGGGAGGTGTAG-3′
*cps 2J*	Forward: 5′-ATGGAAAAAGTCAGCATTATTGTAC-3′
	Reverse: 5′- TTAATCATTATTTTTTTCTTCCCTA-3′
*cps 2K*	Forward: 5′-ATGATTAACATTTCTATCATCGTCC-3′
	Reverse: 5′- TTACCGAGTACTACTTTCACTTCTT-3′
*cps 2L*	Forward: 5′-ATGAATCCAACAAATAGTAGAATAG-3′
	Reverse: 5′- TCAGATGGTTCTCGAATAGCTCAAT-3′
*cps 2M*	Forward: 5′-GTGCGTTCCAAGGTAGATACTTTCA-3′
	Reverse: 5′- TTATCGTTTTCCACGTACTCTCATA-3′
*cps 2N*	Forward: 5′-TTGAGACGAATTTATATTTGCCATA-3′
	Reverse: 5′- CTATTCTATCCCCTCACGCAAAATA-3′
*cps 2P*	Forward: 5′-ATGGTTTATATTATTGCAGAAATTG-3′
	Reverse: 5′- TTACATTTGATTTTCAAAAGCACTA-3′
*cps 2Q*	Forward: 5′-ATGAAAAAAATTTGTTTTGTGACAG-3′
	Reverse: 5′- CTATCTATCATAAAACTCTTTCATG-3′
*cps 2R*	Forward: 5′-ATGAAAAAAGTAGCCTTTCTAGGAG-3′
	Reverse: 5′- CTATTTAATCTTTCTAGCAGGTACA-3′
*cps 2S*	Forward: 5′-ATGGAACCAATTTGTCTGATTCCTG-3′
	Reverse: 5′- TTATCTTGTCAAACTTGTCAAAATC-3′
*cps 2T*	Forward: 5′-ATGAAGCAATTGCTACAGTATTATT-3′
	Reverse: 5′- CTAATAGTATTTTAATATATACTCC-3′
*cps 2U*	Forward: 5′-TTGTTAGTTTATAATTTGGCTAGAG-3′
	Reverse: 5′- TTAAAAAAGGGACTTCAACTTCAAT-3′
*cps 2V*	Forward: 5′-TTATAATGCACTAGTATACTGTATA-3′
	Reverse: 5′- ATGTCTCATTGGAATCAATTTTTAA-3′
*16S rRNA*	Forward: 5′- TGCTAGTCACCGTAAGGCTAAG -3′
	Reverse: 5′- GGCTGCAAGATTTCCTTGAT -3′

### Capsule Extraction, and Capsular Polysaccharide Purification and Isolation

Bacteria were grown in 50 mL of Todd Hewitt broth (THB) at 37°C for 24 h, diluted to 5 L in fresh THB with 1/4 MIC (0.0781 mg/mL) rutin, and cultured for 24 h. Control cells were incubated without rutin. The cells were pelleted by centrifugation at 10000 *g*, suspended in 0.1 mol/L^-1^ Glycine-buffer (Biotopped Ltd, Beijing, China) solution pH 9.2. The CPS was prepared as previously described (Katsumi et al., 1996), with some modifications. Briefly, nucleic acids were removed by precipitation by adding 0.1 mol/L^-1^ CaCl_2_ (Tianli Ltd, Tianjin, China) and ethanol (Tianli Ltd, Tianjin, China) to 25% v/v, and then centrifuged at 7000 *g* at 25°C. The ethanol concentration in the supernatant was enhanced to 80% v/v to precipitate the CPS. The suspension was centrifuged (9000 *g* at 4°C) after overnight at 4 °C. The CPS was purified by gel filtration chromatography on a column filled with Sephacryl S-300 (GE Healthcare, Uppsala, Sweden). The elusion was performed with 50 mmol/L^-1^ NH_4_HCO_3_ (Zhiyuan Ltd, Tianjin, China) at a flow rate of 1.3 mL/min^-1^. Purification was collected and freeze dried. The polysaccharides content was determined by the method (using phenol sulfuric acid) described previously (Dubois et al., 1951).

### The Desialylated Polysaccharide Production

Thirty microgram of sample was hydrolyzed with 0.1 mol/L^-1^ trifluoroacetic acid to determine the composition of monosaccharide (TFA; Zhiyuan Ltd, Tianjin, China) (5 mL) at 80°C for 60 min, and then centrifuged at 10000 *g* for 5 min using ultrafiltration centrifuge tube (Millipore, Billerica, MA, United States). The centrifugation step was repeated three times, and the samples were collected. The top samples are the desialylated polysaccharide, and the bottom samples are the sialic acid. The solution was evaporated to dryness with a stream of N_2_ at room temperature. The precipitation was collected, respectively.

### PMP–HPLC Analysis

Alternatively, in order to determine the composition of monosaccharide, 1 mg of sample was hydrolyzed with 2mol/L^-1^ trifluoroacetic acid (TFA) (0.5 mL) at 120°C for 100 min, at 120°C for 100 min, followed by evaporation with N_2_. Polysaccharide hydrolyzate and monosaccharide standard [glucose (Glc), *N*-acetylglucosamine (GlcN), galactose (Gal), rhamnose (Rha)] (Jinshui Ltd, Shanghai, China) by pre-column derivatization with 1-phenyl-3-methyl-5-pyrazolone (PMP; Sigma, St. Louis, MO, United States). The hydrolysates were fully converted to its PMP derivatives according to the previous method (Guitang et al., 2007), with few modifications. Briefly, the sample was dissolved in 100 μL of H_2_O, and mixed with 100 μL of 0.3 mol/L NaOH (Tianli Ltd, Tianjin, China) and 120 μL of 0.5 mol/L PMP. The mixture was allowed to react for 30 min at 70°C. Then, 100 μL of HCl (0.3 mol/L) (Ligong Ltd, Harbin, Heilongjiang, China) was added to the mixture and 500 μL of chloroform (Ligong Ltd, Harbin, Heilongjiang, China) was added to extract the remaining PMP. The extraction process was repeated three times. The PMP derivatized sample solution was filtrated through a 0.22 μm membrane. Then the compositions of monosaccharide were determined by HPLC (Waters, Shanghai, China) with an UV detector at 245.0 nm on a Waters chromatograph. The mobile phase was made of Methanol (Kemiou Ltd, Tianjin, China) (A) and 0.01 mol/mL formic acid (Kemiou Ltd, Tianjin, China) (pH 6.8) (B) (A:B = 82:18, v/v). With 20 μL of injection volume, separation was performed on a Spherisorb-C18 Column (Waters, Shanghai, China) (5 μm, 250 mm × 4.6 mm) at 30°C and 1 mL/min of flow rate.

### Analysis of Sialic Acid by the Fluorometric HPLC Methods

The sialic acid samples and *N*-acetylgucosamine acid (Neu5Ac; Sigma, St. Louis, MO, United States) were added 0.2 mL of 0.01 mol/L^-1^ TFA and 0.2 mL of 7 mmol/L^-1^ DMB (TCI, Tokyo, Japan) solution in 5 mmol/L trifluoroacetic acid containing 1 mol/L 2-mercaptoethanol and 18 mmol/L sodium hydrosulfite (Guangfu, Tianjin, China), and incubated at 60°C for 2 h. The sample solution filtrated through a 0.22 μm membrane after derivatized by DMB. The composition of monosaccharide was determined by HPLC equipped with an EV detector (Ex 373 nm and Em 448nm) on a Waters chromatographic system. The mobile phase was made of CH_3_OH (A), CH_3_CN (Kemiou Ltd, Tianjin, China) (B) and 0.05% TFA (C) (A: B: C = 6:4:90, v/v/v). Separation was performed on a Spherisorb-C18 Column (5 μm, 250 mm × 4.6 mm) at 26°C and 1 mL/min of flow rate at an injection volume of 20 μL.

### Nuclear Magnetic Resonance

The polysaccharide and the desialylated polysaccharide were exchanged in 33 mmol/L^-1^ phosphate buffer (pH 8.0) in ^2^H_2_O (99.9 atom% ^2^H), freeze dried, and dissolved in the same amount of ^2^H_2_O (99.96 atom% ^2^H). Nuclear magnetic resonance (Bruker, Beijing, China, NMR) spectra were acquired on polysaccharide samples at concentrations of circa (1%–3%).

### Statistical Analysis

All the assays were performed in triplicate, and the results were expressed as means ± standard deviations. Data were analyzed by the Student’s *t-*test. Statistics were determined using SPSS software, version 18.0.

## Results

### Effectiveness of Rutin on Inhibition of Biofilm Formation by the TCP Assay

**FIGURE 1:**
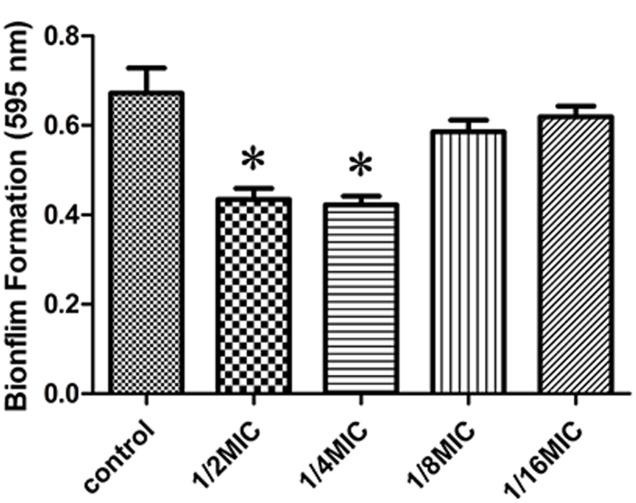
Effect of rutin at different concentrations on *Streptococcus suis* biofilm formation. The data are expressed as the means ± standard deviations. Asterisk indicates statistically significant difference of treatment versus untreated culture (^∗^*p* < 0.05).

We determined the effect of sub-inhibitory concentrations of rutin on biofilm production *in vitro*. The MIC values of rutin for *S. suis* was 0.3125 μg⋅mL^-1^. As shown in **Figure 1**, 1/2 MIC (0.1563 mg/mL) and 1/4 MIC (0.0781 mg/mL) of rutin were able to significantly inhibit biofilm production (*p* < 0.05). Rutin at 1/8 MIC (0.0391 mg/mL) and 1/16 MIC (0.0195 mg/mL) had no significant effect on *S. suis* biofilm formation.

### Growth Inhibitory Activity of Rutin

**FIGURE 2:**
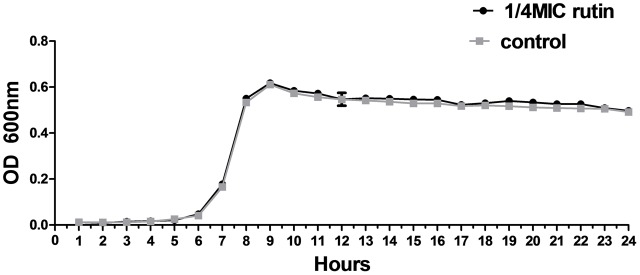
Growth curve of *S. suis* in the absence of rutin and in the presence of rutin at 1/4 MIC (0.0781 mg/ml). The data are expressed as the mean ± standard deviations.

The growth of *S. suis* was measured for cultures with 0 MIC and 1/4 MIC (0.0781 mg/mL) of rutin. The growth curves for 1/4 MIC of rutin were not significantly different in lag, exponential, and stationary phases during 24 h of incubation (**Figure 2**). The results suggested that the growth of *S. suis* was unaffected by the addition of 1/4 MIC (0.0781 mg/mL) rutin.

### Direct Observation of Biofilm Formation *In Vitro* by SEM

**FIGURE 3:**
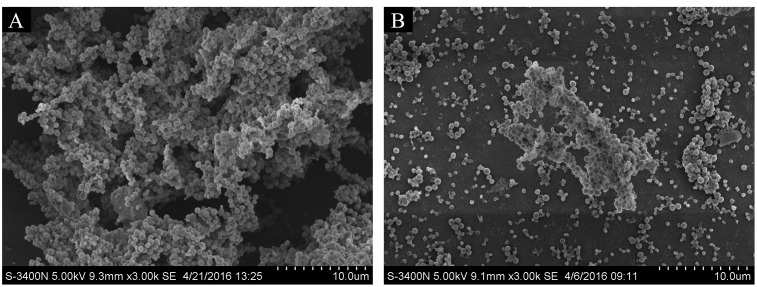
Effect of rutin on *S. suis* biofilm by scanning electron microscope. Biofilm formation of *S. suis* without rutin **(A)**. Biofilm formation of *S. suis* with 1/4 MIC (0.0781 mg/ml) rutin treatment **(B)**.

To observe the effect of rutin on biofilm production, the architecture of biofilms formed in the absence or in the presence 1/4 MIC (0.0781 mg/mL) of rutin was studied by SEM. As shown in **Figure 3A**, when biofilm was formed in the absence of rutin, the aggregates and microcolonies of *S. suis* covered almost entirely the glass slide surface. However, when biofilm was formed in the presence 1/4 MIC (0.0781 mg/mL) of rutin, a marked variability in the three-dimensional biofilm architecture was noted. Biofilm thickness is thin and bacteria are scattered (**Figure 3B**). These results suggested that rutin at 1/4MIC (0.0781 mg/mL) significantly inhibited the biofilm formation of *S. suis in vitro.*

### Anti-adherence Activity of Extract Against *S. suis*

**FIGURE 4:**
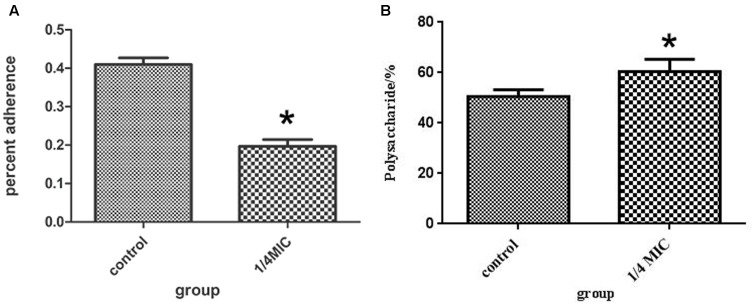
Effect of 1/4 MIC (0.0781 mg/ml) rutin on *S. suis* adhesion **(A)**. Effect of 1/4 MIC (0.0781 mg/ml) rutin on the polysaccharides content of *S. suis* **(B)**. The data are expressed as means ± standard deviations. Asterisk indicates statistically significant difference of treatment versus untreated culture (^∗^*p* < 0.05).

The inhibitory effects at 1/4 MIC (0.0781 mg/mL) of rutin on adherence of *S. suis* are shown in **Figure 4A**. The rutin inhibited adherence in a pronounced manner. The anti-adherence rate of *S. suis* is 20% at a concentration of 1/4MIC (0.0781 mg/mL) of rutin.

### PCR Amplification and Nucleotide Sequencing

The mapping of the *cps* genes in the genome of *S. suis* by PCR are shown in **Figure 5**. Sequencing was performed to confirm that no mutation occurred. Detailed information is shown in the **Supplementary Figures S1**–**S21**. The **Supplementary Figures S1**–**S21** shows the results of *cps2A-cps2U* sequence alignment. The sequence of the *cps* gene cluster has no difference between rutin-treated and untreated cells.

**FIGURE 5:**
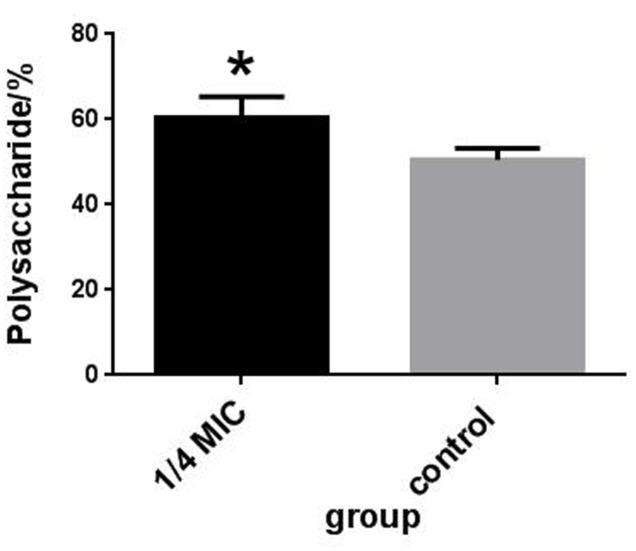
Mapping of the *cps* in the genome of *S. suis* by PCR. The 1–21 stands for *cps2A–2U*, respectively.

### The Effect of 1/4 MIC of Rutin on Expression of *cps* Genes

**FIGURE 6:**
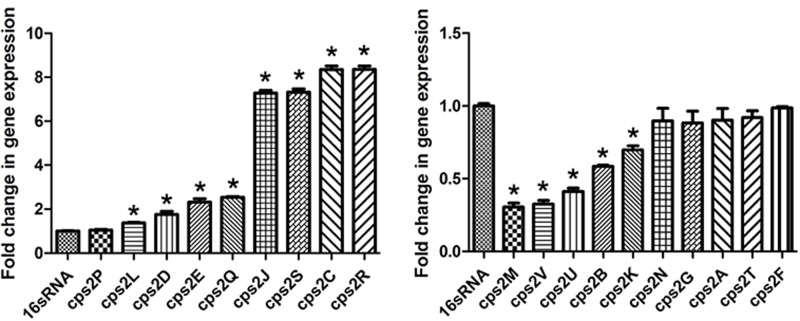
Effect of rutin on mRNA expression of *cps* genes in *S. suis*. The data are expressed as means ± standard deviations. The expression was normalized to 16S rRNA. Controls refer to the absence of rutin. Asterisk indicates statistically significant difference of treatment versus untreated culture (^∗^*p* < 0.05).

Thereafter, the effect of rutin on the expression profile of *cps* gene cluster was analyzed. As displayed in **Figure 6**, when the culture medium was in the presence 1/4 MIC (0.0781 mg/mL) of rutin, expression of the genes *cps2C, cps2D, cps2E, cps2J, cps2L, cps2R*, and *cps2Q* was up regulated. However, the expression of the genes *cps2B, cps2M, cps2K, cps2U*, and *cps2V* was down regulated.

### Capsule Polysaccharide Content Determination

The rutin effect for polysaccharides content of *S. suis* was shown in **Figure 4B**. The contents of total sugar were analyzed by phenol-sulfuric method. 50.54 ± 2.69 % and 60.46 ± 4.91 % of the polysaccharides content were observed in the control and rutin group, respectively (*P* < 0.05). The results suggested that the polysaccharides content significantly increased when the bacteria was incubated with rutin.

### HPLC Characterization of Polysaccharides

**FIGURE 7:**
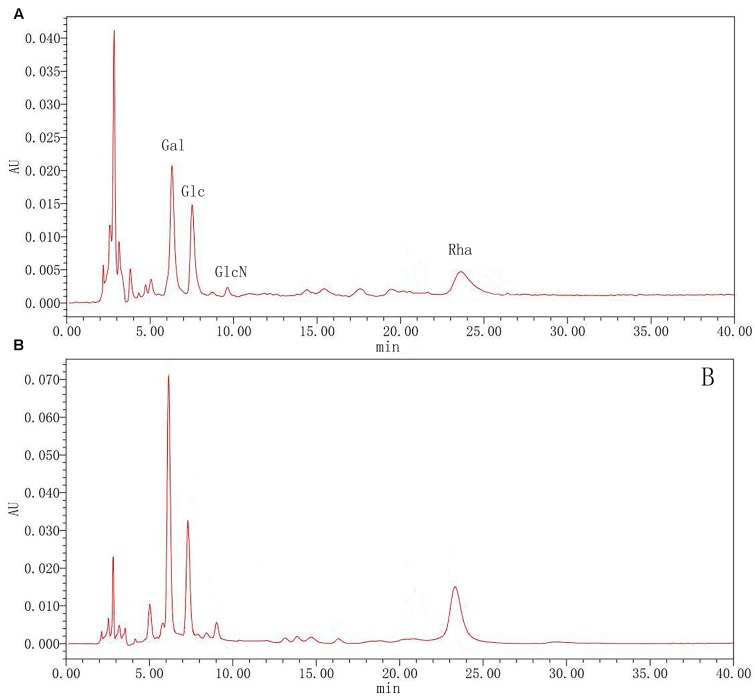
High performance liquid chromatography (HPLC) chromatograms of monosaccharides after hydrolysis of polysaccharide in control **(A)** and rutin **(B)** groups.

To further clarify polysaccharides, the compositions were examined by acid hydrolysis and HPLC. After PMP derivatization, the composition of monosaccharides from the hydrolyzed products was examined by HPLC. From the chromatograms, Glc and Gla, GlcN and Rha were detected in both rutin group and control group. As shown in **Figure 7**, the results showed that there was the no significant difference between the rutin and control group. The molar ratio of monosaccharides (Rha: Gal: Glc: GlcN) was 1:2.22:0.95:0.76 and 1: 2.03: 1.29: 0.74 for the rutin group and control group, respectively. These results exhibited that the monosaccharide compositions had no significant difference when the culture medium was added with 1/4 MIC (0.0781 mg/mL) rutin.

**FIGURE 8:**
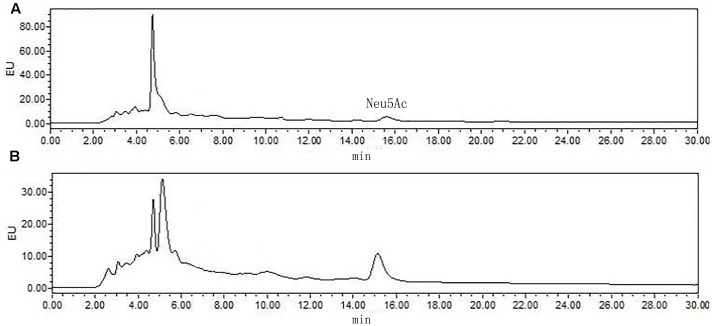
High performance liquid chromatography chromatograms of sialic acid (Neu5Ac) in control **(A)** and rutin **(B)** groups.

The most significant feature of the sialic acid is their instability. DMB possess significant advantages in its specific reactivity with the α-keto acids of sialic acids. After DMB derivatization, the sialic acid from the hydrolyzed products was determined by HPLC. As shown in **Figure 8**, Neu5Ac was detected in the product of the rutin group and the control group. The ratio of sialic acid content of the control and rutin group was 1:1.84. The results of HPLC suggested that the content of Rha, Glc, Gal, GlcN, and Neu5Ac of rutin group was generally higher than that of control group.

### Nuclear Magnetic Resonance

**FIGURE 9:**
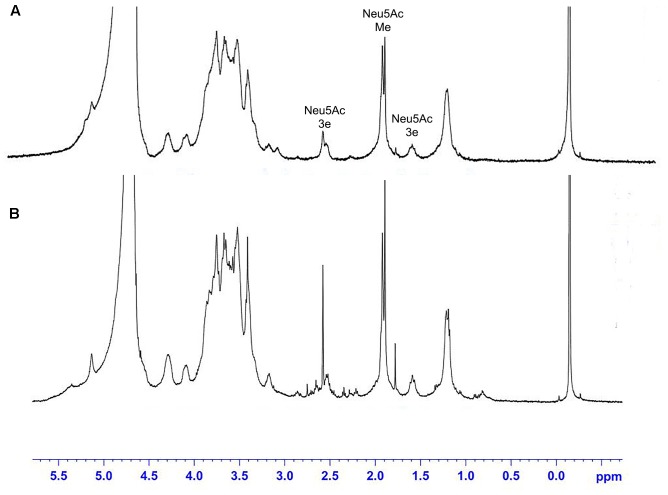
500 MHz ^1^H nuclear magnetic resonance (NMR) spectra of capsular polysaccharide in control **(A)** and rutin **(B)** groups.

**FIGURE 10:**
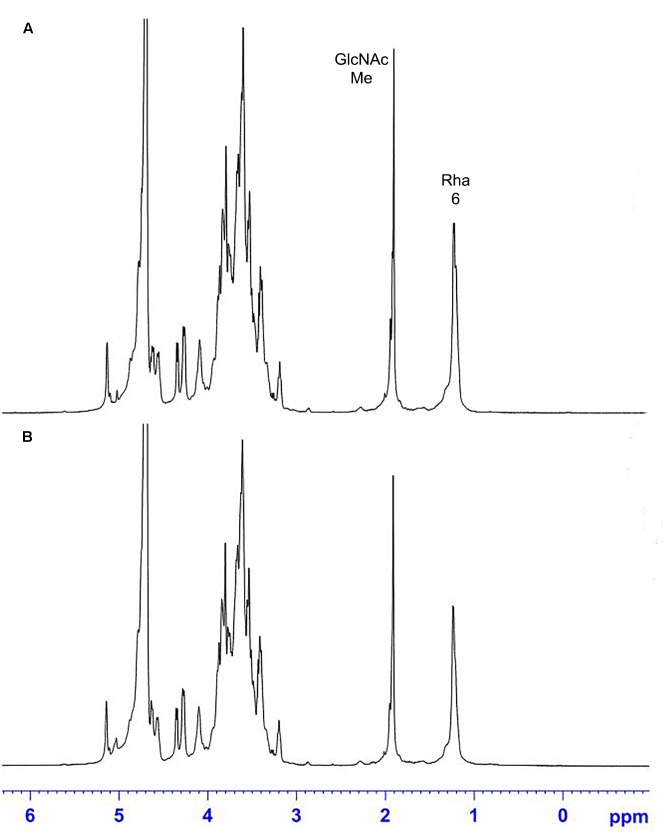
500 MHz ^1^H NMR spectra of desialylated polysaccharide in control **(A)** and rutin **(B)** groups.

The ^1^H NMR spectra of the *S. suis* CPS of rutin group and control group before and after desialylated are shown in **Figures 9**, **10**. The same spectra was observed in the ^1^H NMR spectra of the *S. suis* CPS after and before acid hydrolysis reported by the Van Calsteren (Van Calsteren et al., 2010). The ^1^H NMR spectra of the polysaccharides of *S. suis* of control group and rutin group are shown in **Figure 9**. There was conformity between two polysaccharides for their ^1^H NMR spectra. The ^1^H NMR spectra of the desialylated polysaccharides of *S. suis* control group and rutin treated group are shown in **Figure 10**. The result showed that there was no difference between the structures of two desialylated polysaccharide. On the ^1^H NMR spectra, polysaccharide signals were concentrated in the δ 3.3 ∼ 4.0 ppm range. Reporter resonance signals were identified on both spectra: acetyl methyl protons of *N*-acetylglucosamine near δ 2.0, and methyl protons in position 6 of rhamnose at crica δ 1.2. Signals characteristic of sialic acid, that is, acetyl methyl protons near δ 2.0 and methylene protons in position 3 at δ 2.579 and 1.591 vanished fully after hydrolysis. The only difference between the spectra of the **Figures 9**, **10** was the loss of the sialic acid signals.

## Discussion

In this work the ability of rutin to inhabit *S. suis* biofilms was investigated by TCP assay and SEM. The TCP assay according to the ability of bacteria to form biofilms on the microplates indicated indirectly that the sub-inhibitory concentrations of rutin could inhibit the *S. suis* biofilm formation *in vitro*. Then, the effect of rutin on the structure of biofilm of *S. suis* was observed directly by SEM. Our results showed that rutin could inhibit *S. suis* biofilm formation at 1/4MIC (0.0781 mg/mL). However, it is still unclear why rutin affects *S. suis* biofilms *in vitro*. In addition, a question remained unanswered: how does rutin affect *S. suis* biofilms at the molecular level? Then, we investigated the influence of rutin on *S. suis* growth *in vitro*. The results suggested that rutin could significantly inhibit biofilm formation of *S. suis* but had had no effect on its growth by the addition of 1/4MIC (0.0781 mg/mL) rutin. Rutin was previously shown to affect attachment of *Staphylococcal aureus* (Liu et al., 2015) and inhibit the *Pseudomonas aeruginosa* biofilm formation (Lou et al., 2015). Thus, anti-adherence activity of rutin was investigated. Our results showed that rutin inhibited adherence in an obvious manner.

Previous reports had demonstrated that CPS was play an important role in the adhesion of host cells (Allegrucci and Sauer, 2007), and the encapsulated clinical *pneumococcal* isolates have impaired biofilm formation (Moscoso et al., 2006). The structure of the CPS can also affect biofilm formation. For example, the α-D-Glc is partially replaced with α-D-GlcNAc in *Vibrio cholera* polysaccharide which gives it high viscosity and promotes biofilm formation (Yildiz et al., 2014). Thus, the content and structure of *S. suis* CPS were detected. Results showed that rutin are beneficial to improve the CPS content of *S. suis.* Elliott and Tai (Elliott and Tai, 1978) defined the capsular polysaccharide of *S. suis* as consisting of five sugars including Rha, Glc, Gal, GlcNAc and Neu5Ac. The polysaccharides in the rutin and control group contained five monosaccharides as above after hydrolysis and derivation. It is consistent with the results of Elliott and Tai (1978). Van Calsteren has given the description about the sugar structure of *S. suis* CPS (Van Calsteren et al., 2010). In this study, the same ^1^H NMR spectra was observed in *S. suis* CPS when *S. suis* was cultivated in the presence of rutin. Our findings are in consistent with the results of Van Calsteren (Van Calsteren et al., 2010). According to our results, rutin had no effect on the structure of *S. suis* CPS. Therefore, rutin may affect the capsular polysaccharide content and then affect the bacterial adhesion and biofilm formation.

Finally, we addressed the question that how rutin resulted in an increased content of *S. suis* capsular polysaccharide. It has been stated earlier that multiple genes involved in the synthesis of CPS. Smith et al. (1999) identified *cps* gene cluster which is essential for the synthesis of the repeating unit of CPS and found that CPS is comprised of Glc, Gal, GlcN, Rha, and Neu5Ac. Van Calsteren et al. (2010) elucidated CPS structure of *S. suis* and predicted the *cps* genes responsible for the synthesis of CPS repeating units. In addition, *S. suis* CPS were considered to be synthesized by the *wzx*/*wzy*-dependent pathway due to possession of *wzx* and *wzy* genes (Wang K. et al., 2011; Okura et al., 2013). The genes in *wzx*/*wzy*-dependent pathway comprise of *cps* gene cluster and are usually located at the same locus on chromosome (Bentley et al., 2006).

It has been predicted previously that *cps2E* gene was the initiating gene in the process of the synthesis of the repeating unit of CPS, and the removal of *cps2E* gene led to the complete absence of CPS, as was previously reported in *Streptococcus pneumoniae* (Cartee et al., 2005). In our study, we found that *cps2E* gene was significantly upregulated in the presence of rutin. The up regulation of *cps2E* may promote the synthesis of the capsular polysaccharide and thus inhibit biofilm formation. It has been demonstrated that the gene *cps2L* was responsible for the synthesis of sialyltrans-ferase which in turn mediate the transfer of sialic acid to CPS repeating units (Van Calsteren et al., 2010). The sialic acid played a major role in the regulation of bacterial adhesion (Charland et al., 1996; Segura and Gottschalk, 2002; Lewis et al., 2012). The expression level of *cps2L* gene was up regulated in the present study. The up regulation of *cps2L* may promote the synthesis of the capsular polysaccharide.

Previously, it has been predicted that the gene *cps2G* and *cps2J* were involved in the synthesis of long side chain and short side chain of *cps* units (Van Calsteren et al., 2010). Although the expression level of *cps2G* gene did not change, the expression level of *cps2J* gene was up regulated in the present study. Furthermore, *cps2L*, *cps2E*, *cps2J*, and *cps2G* gene were found in *S. suis* responsible for different glycosyltransferases involved in CPS synthesis. The above four deletion mutants with their sialic acid content decreased and CPS incomplete in *S. suis* SC19 and indicated enhanced adhesion to host cells (Zhang et al., 2016). Therefore, the up regulation of *cps2J* may promote the synthesis of the capsular polysaccharide.

It has been demonstrated that CpsD is an autophosphorylating tyrosine kinase regulated by CpsC that is required for CpsD tyrosine phosphorylation (Morona et al., 2000; Bender and Yother, 2001) and both may be important regulators connecting capsule synthesis to cell division. CpsB is a manganese dependent tyrosine phosphatase that influences dephosphorylation of CpsD, as a kinase inhibitor, autophosphorylation of CpsB may prevent CpsD in its C-terminal tyrosine and weaken the activity of the CpsD protein, and inhibit the production of capsular polysaccharide. It has been mentioned earlier that a *cps2D*-deleted strain produced lesser amount of CPS was avirulent (Morona et al., 2004). Much higher expressions were detected for *cps2C* and *cps2D* when *S. suis* was cultivated in the presence of rutin. The expression level of *cps2A and cps2B* gene was down regulated in the present study. *cpsB*, *cpsC*, and *cpsD* are essential for encapsulation, while *cpsA* is not necessary as previously reported (Morona et al., 2004). So, the expression level of *cps2A* gene down regulated may have no effect on the amount of capsular polysaccharide. These data suggested that the *cps2B*, *cps2C*, and *cps2D* expression might be partly responsible for the reduced biofilm. However, the detailed molecular mechanism still unknown.

## Conclusion

Our findings demonstrate that 1/4MIC (0.0781 mg/mL) of rutin inhibits *S. suis* biofilm formation without impairing its growth *in vitro*. We investigate the effect of rutin on *S. suis* CPS content and structure. The results reveal that rutin is beneficial to improve the polysaccharide content of *S. suis* without changing its structure. The potential mechanism may be that rutin is beneficial to improve the polysaccharide content of *S. suis* which affects bacterial adhesion. The *cps2B*, *cps2C*, *cps2D*, *cps2E*, *cps2J*, and *cps2L* might be responsible for the reduced biofilm. Altogether our findings demonstrate that rutin interfers with CPS synthesis in *S. suis* and thereby decreases biofilm formation of *S. suis*. Thus, our findings found the rough regulation of *S. suis* biofilm formation that may be utilized potentially to manage *S. suis* biofilm infections.

## Author Contributions

Conceived and designed the experiments: YL. Performed the experiments: SW, CW, LG, YZ, YY, CX. Analyzed the data: WD, JC, IM, XC. Contributed reagents/materials/analysis tools: HC, XH, DL.

## Conflict of Interest Statement

The authors declare that the research was conducted in the absence of any commercial or financial relationships that could be construed as a potential conflict of interest.

## Supplementary Material

FIGURE S1–S21 Effect of rutin on cps gene sequences.
