# Supplementary figures and images for: Rutin Inhibits Streptococcus suis Biofilm Formation by Affecting CPS Biosynthesis
Source: Front Pharmacol. 2017 Jun 16;8:379. doi: 10.3389/fphar.2017.00379 (PMC5472726; doi:10.3389/fphar.2017.00379)

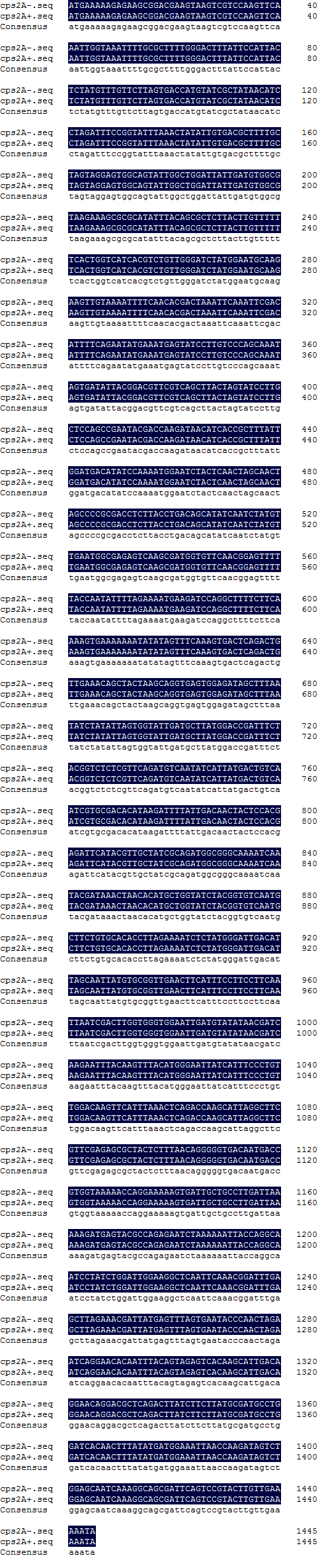

Supplement: FIGURE S1–S21 — Effect of rutin on cps gene sequences. [file Presentation_1.ZIP › supplementary file/cps2A.tif]

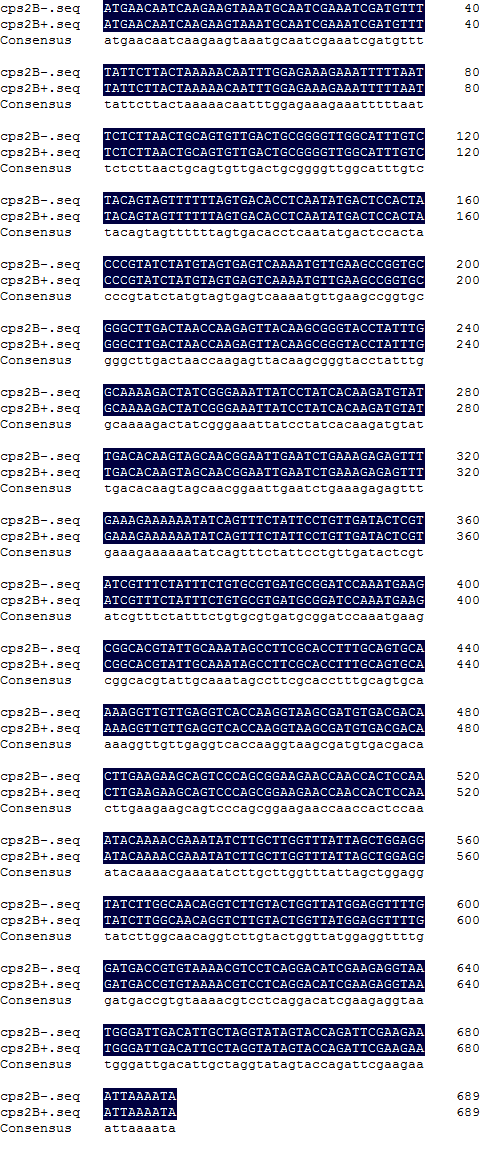

Supplement: FIGURE S1–S21 — Effect of rutin on cps gene sequences. [file Presentation_1.ZIP › supplementary file/cps2B.tif]

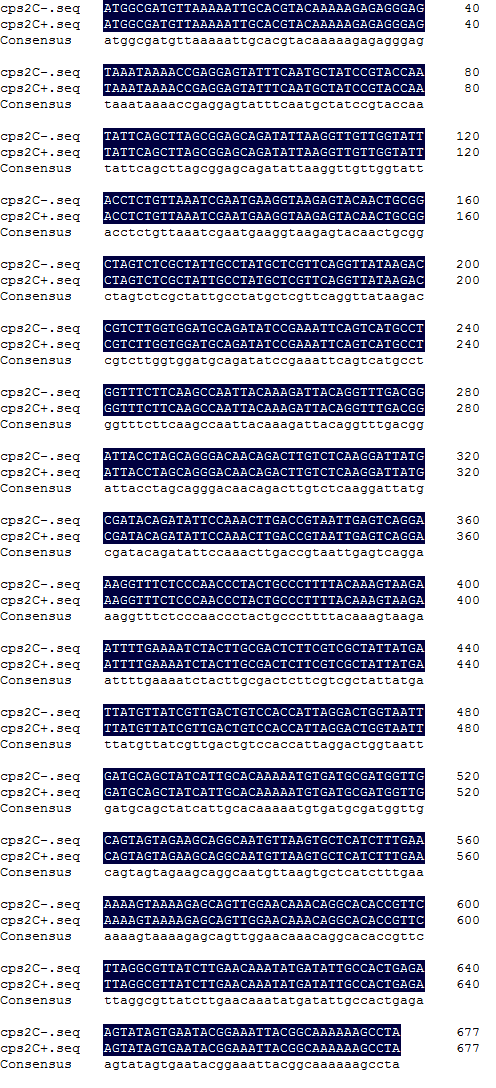

Supplement: FIGURE S1–S21 — Effect of rutin on cps gene sequences. [file Presentation_1.ZIP › supplementary file/cps2C.tif]

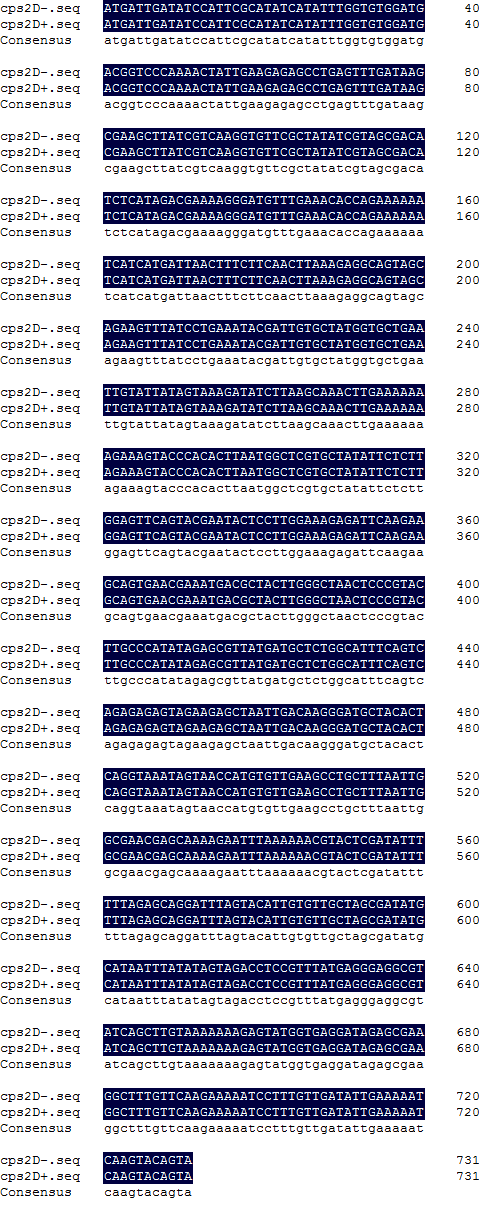

Supplement: FIGURE S1–S21 — Effect of rutin on cps gene sequences. [file Presentation_1.ZIP › supplementary file/cps2D.tif]

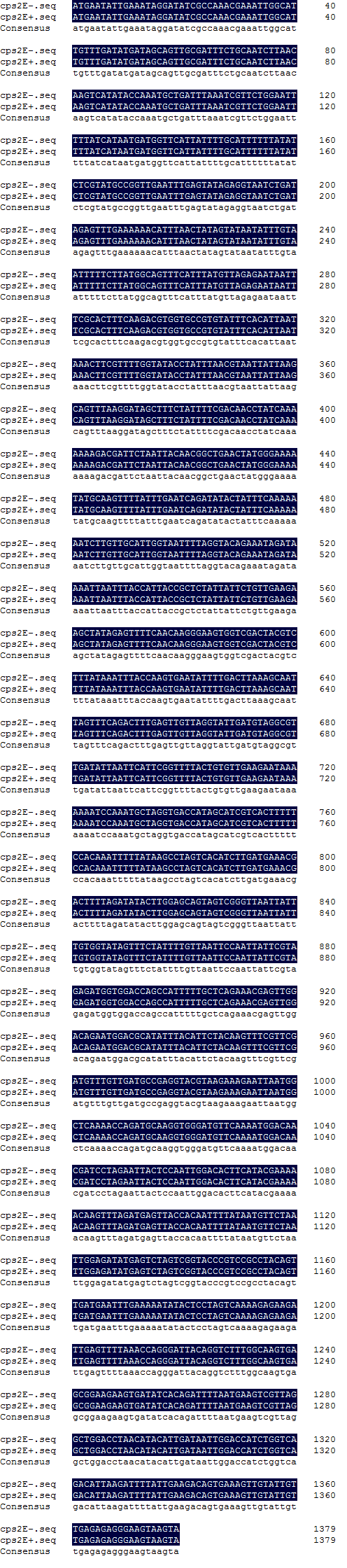

Supplement: FIGURE S1–S21 — Effect of rutin on cps gene sequences. [file Presentation_1.ZIP › supplementary file/cps2E.tif]

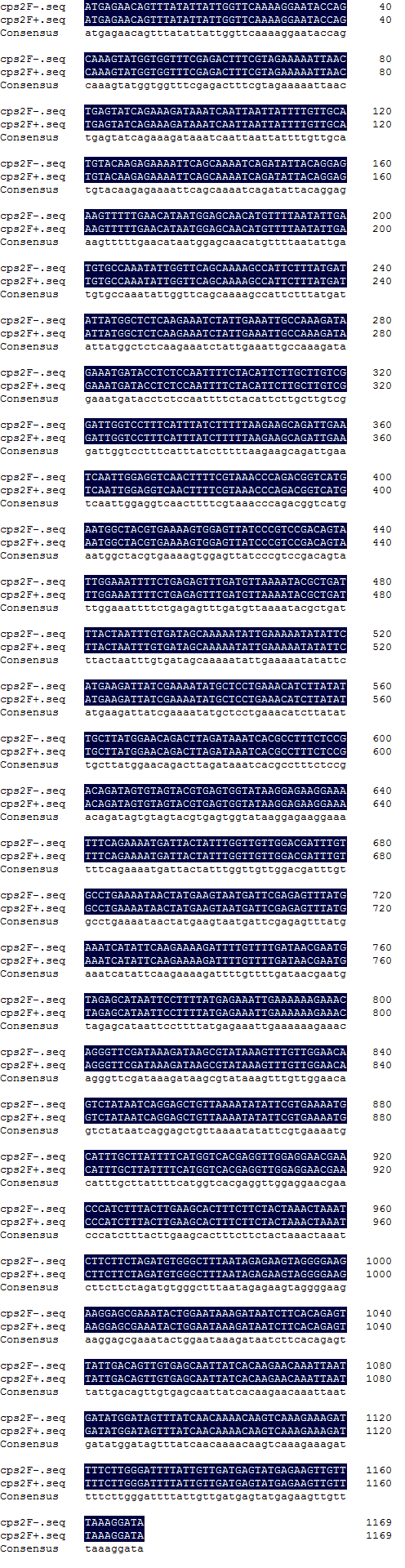

Supplement: FIGURE S1–S21 — Effect of rutin on cps gene sequences. [file Presentation_1.ZIP › supplementary file/cps2F.tif]

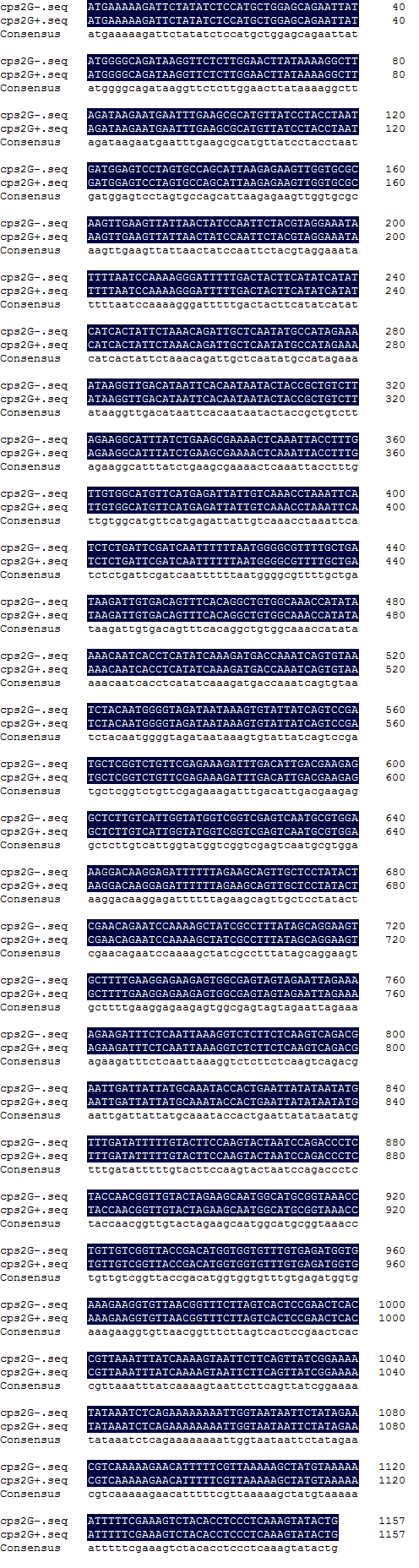

Supplement: FIGURE S1–S21 — Effect of rutin on cps gene sequences. [file Presentation_1.ZIP › supplementary file/cps2G.tif]

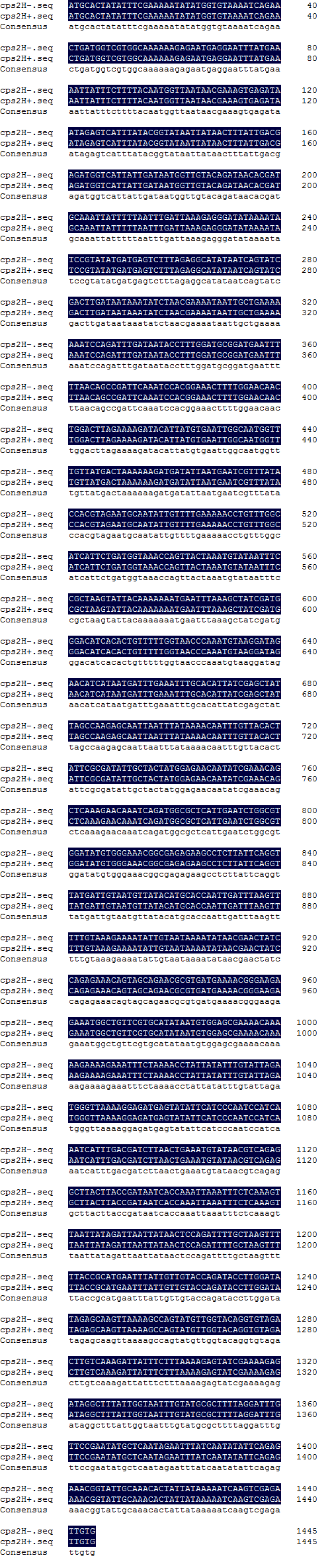

Supplement: FIGURE S1–S21 — Effect of rutin on cps gene sequences. [file Presentation_1.ZIP › supplementary file/cps2H.tif]

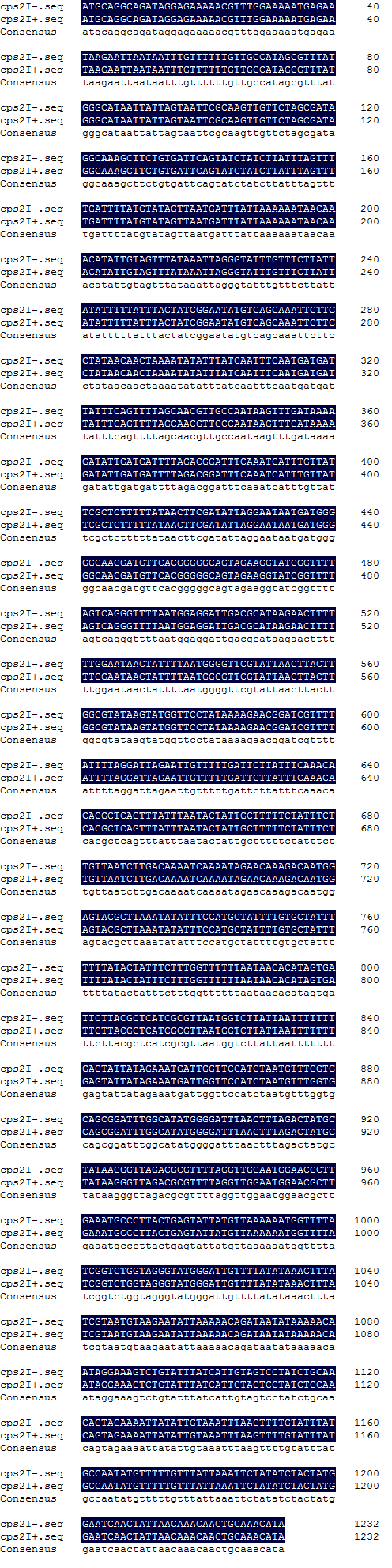

Supplement: FIGURE S1–S21 — Effect of rutin on cps gene sequences. [file Presentation_1.ZIP › supplementary file/cps2I.tif]

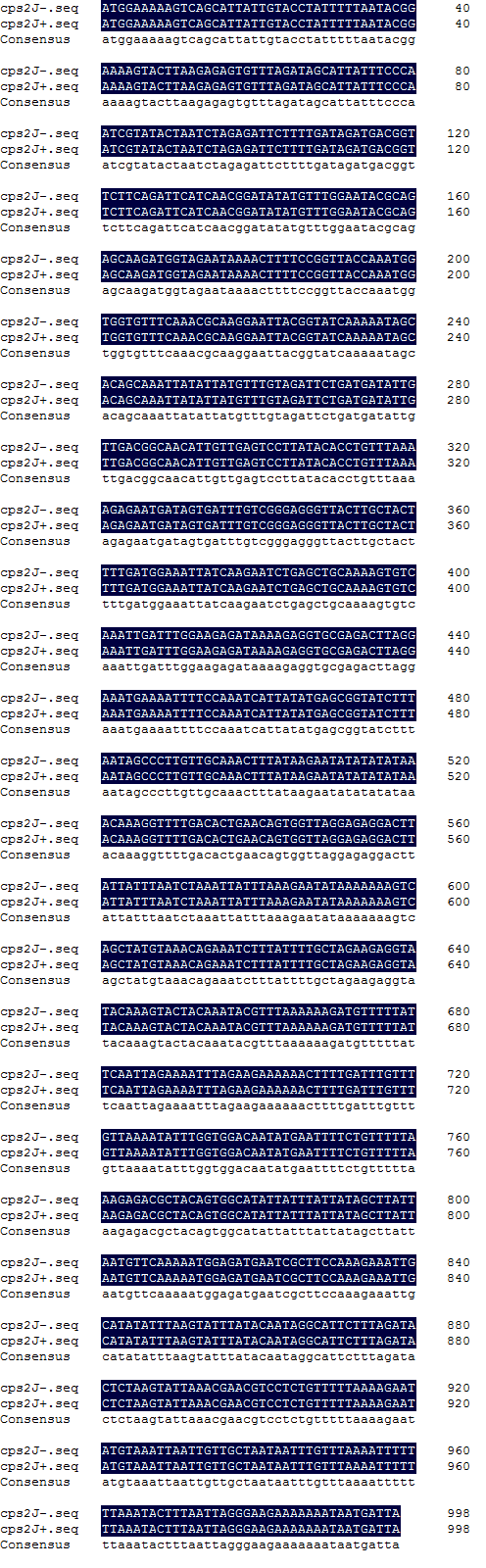

Supplement: FIGURE S1–S21 — Effect of rutin on cps gene sequences. [file Presentation_1.ZIP › supplementary file/cps2J.tif]

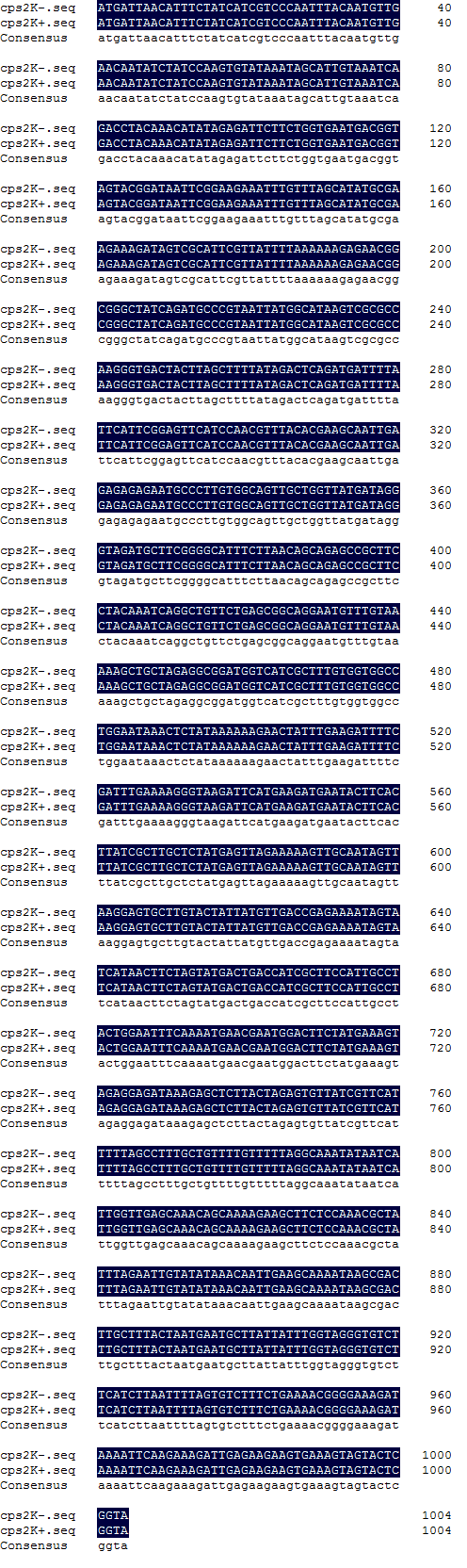

Supplement: FIGURE S1–S21 — Effect of rutin on cps gene sequences. [file Presentation_1.ZIP › supplementary file/cps2K.tif]

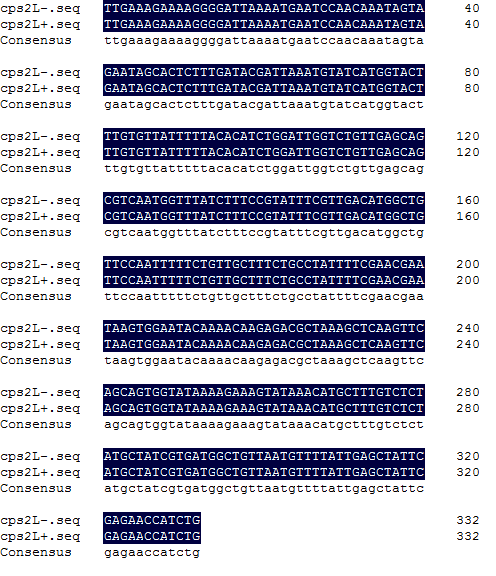

Supplement: FIGURE S1–S21 — Effect of rutin on cps gene sequences. [file Presentation_1.ZIP › supplementary file/cps2L.tif]

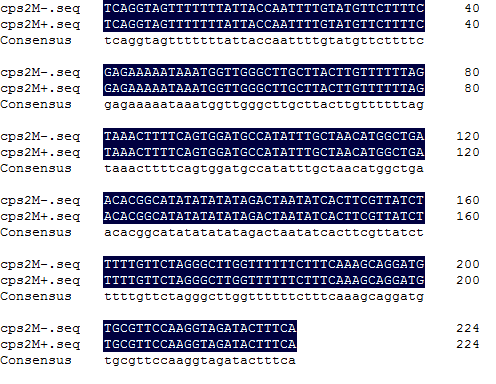

Supplement: FIGURE S1–S21 — Effect of rutin on cps gene sequences. [file Presentation_1.ZIP › supplementary file/cps2M.tif]

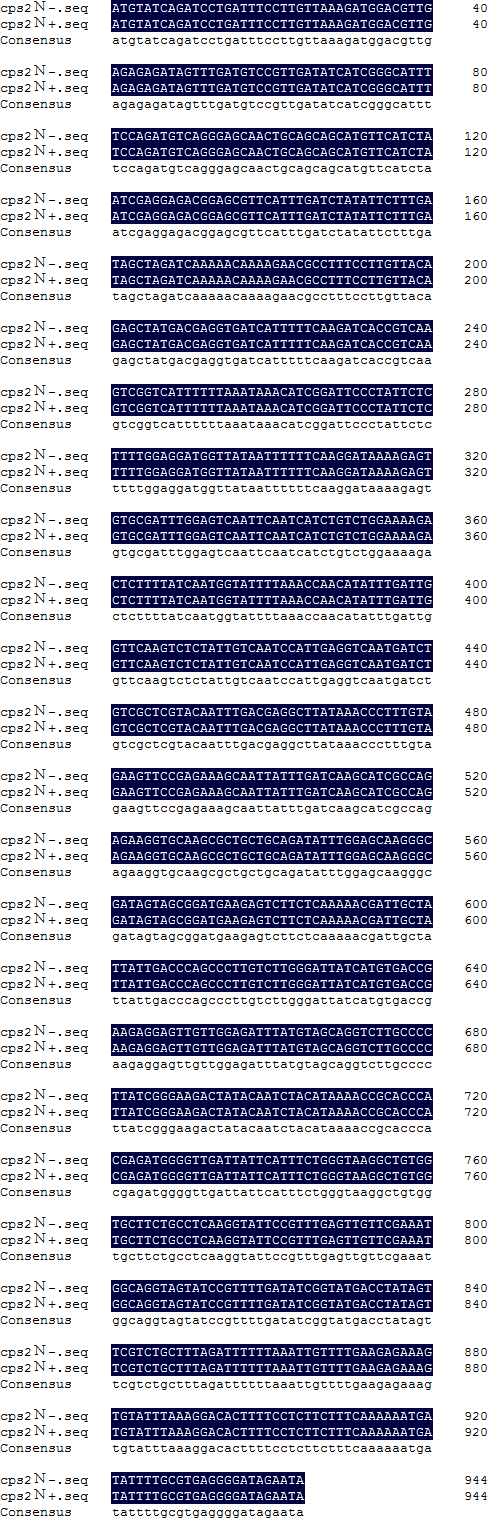

Supplement: FIGURE S1–S21 — Effect of rutin on cps gene sequences. [file Presentation_1.ZIP › supplementary file/cps2N.tif]

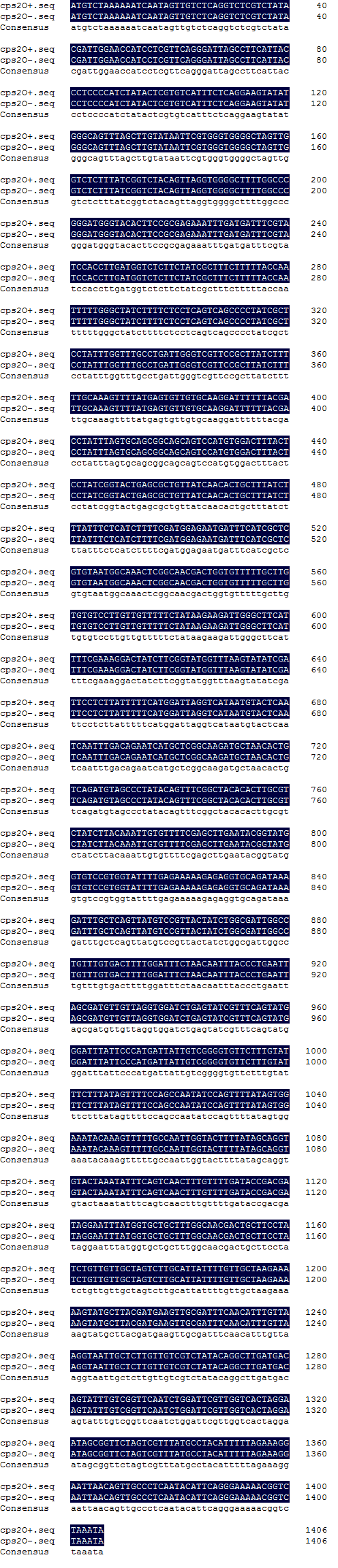

Supplement: FIGURE S1–S21 — Effect of rutin on cps gene sequences. [file Presentation_1.ZIP › supplementary file/cps2O.tif]

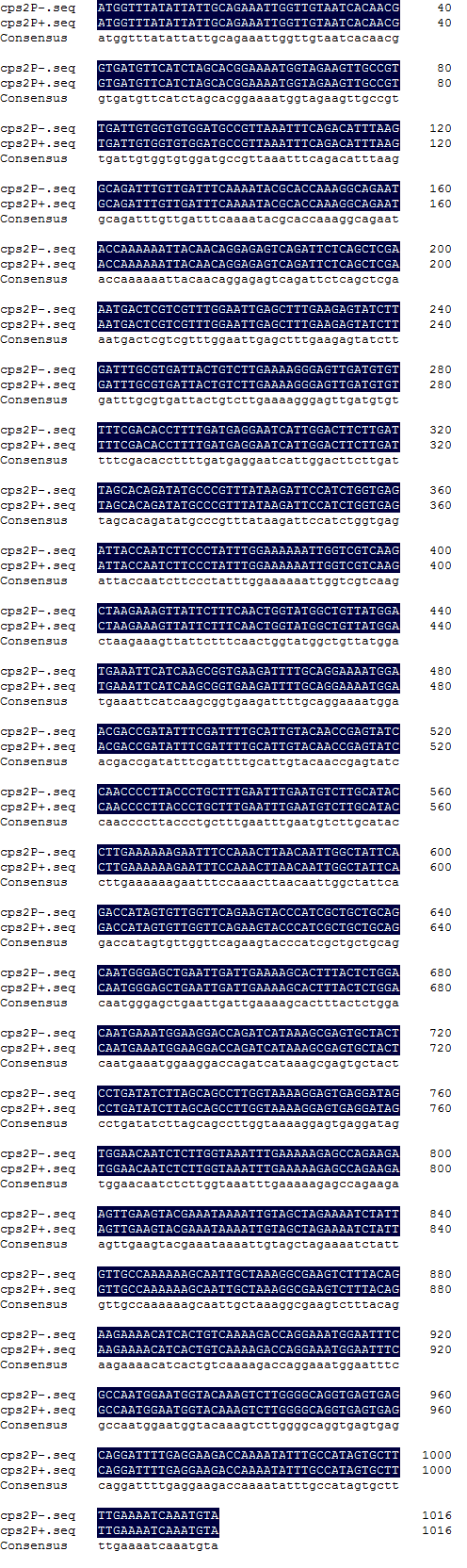

Supplement: FIGURE S1–S21 — Effect of rutin on cps gene sequences. [file Presentation_1.ZIP › supplementary file/cps2P.tif]

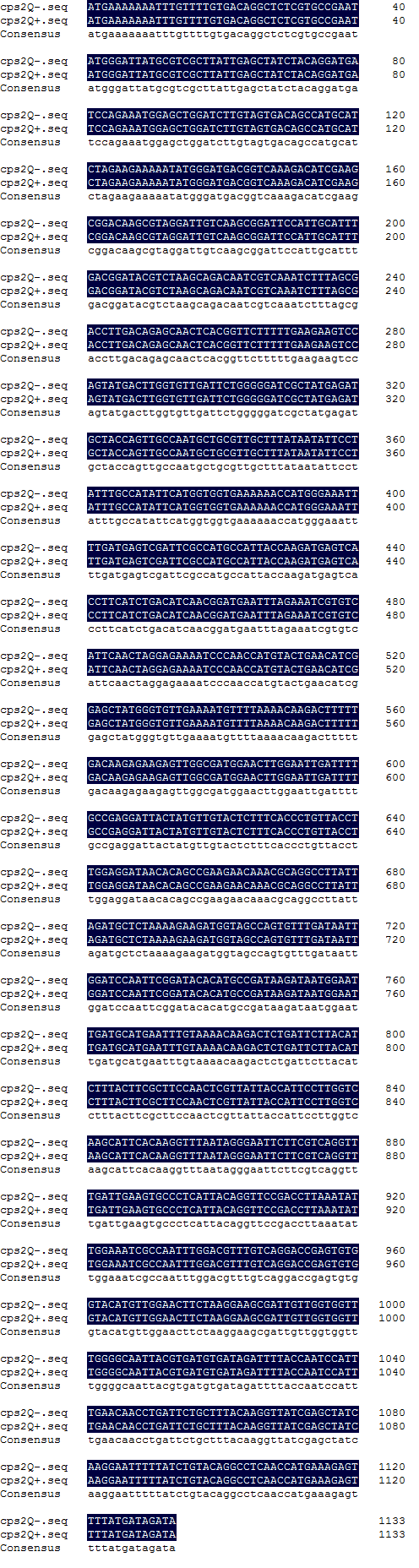

Supplement: FIGURE S1–S21 — Effect of rutin on cps gene sequences. [file Presentation_1.ZIP › supplementary file/cps2Q.tif]

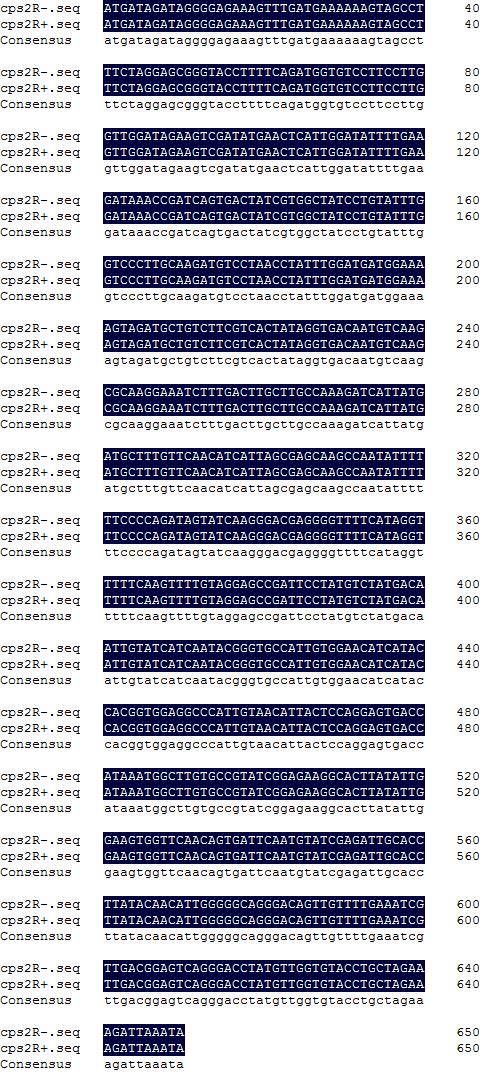

Supplement: FIGURE S1–S21 — Effect of rutin on cps gene sequences. [file Presentation_1.ZIP › supplementary file/cps2R.tif]

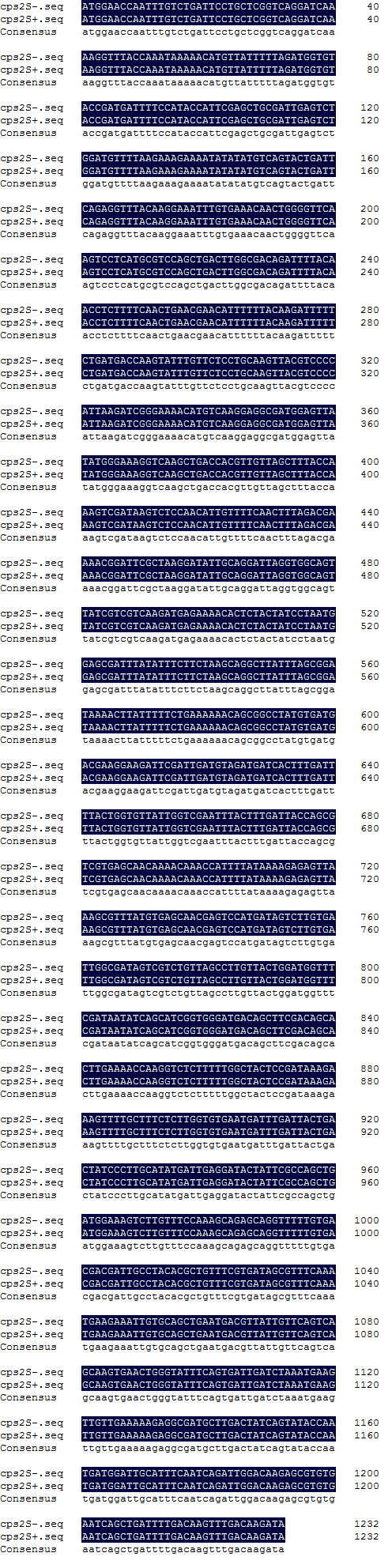

Supplement: FIGURE S1–S21 — Effect of rutin on cps gene sequences. [file Presentation_1.ZIP › supplementary file/cps2S.tif]

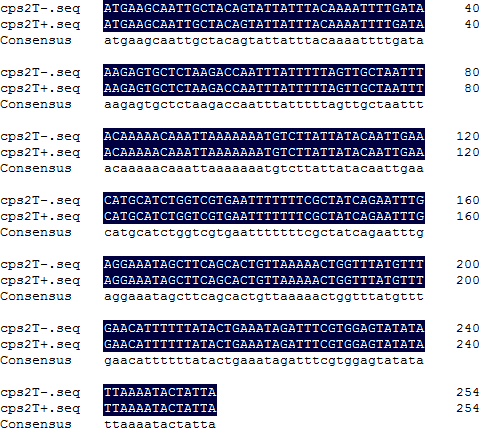

Supplement: FIGURE S1–S21 — Effect of rutin on cps gene sequences. [file Presentation_1.ZIP › supplementary file/cps2T.tif]

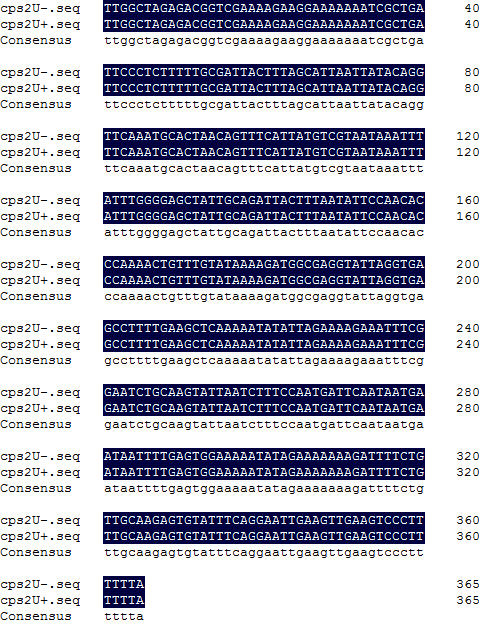

Supplement: FIGURE S1–S21 — Effect of rutin on cps gene sequences. [file Presentation_1.ZIP › supplementary file/cps2U.tif]
